# Different Degrees of Plant Invasion Significantly Affect the Richness of the Soil Fungal Community

**DOI:** 10.1371/journal.pone.0085490

**Published:** 2013-12-31

**Authors:** Chuncan Si, Xueyan Liu, Congyan Wang, Lei Wang, Zhicong Dai, Shanshan Qi, Daolin Du

**Affiliations:** 1 School of the Environment and Safety Engineering, Jiangsu University, Zhenjiang, Jiangsu, China; 2 Key Laboratory of Modern Agricultural Equipment and Technology, Ministry of Education and Jiangsu Province, Jiangsu University, Zhenjiang, Jiangsu, China; Beijing Forestry University, China

## Abstract

Several studies have shown that soil microorganisms play a key role in the success of plant invasion. Thus, ecologists have become increasingly interested in understanding the ecological effects of biological invasion on soil microbial communities given continuing increase in the effects of invasive plants on native ecosystems. This paper aims to provide a relatively complete depiction of the characteristics of soil microbial communities under different degrees of plant invasion. Rhizospheric soils of the notorious invasive plant *Wedelia trilobata* with different degrees of invasion (uninvaded, low-degree, and high-degree using its coverage in the invaded ecosystems) were collected from five discrete areas in Hainan Province, P. R. China. Soil physicochemical properties and community structure of soil microorganisms were assessed. Low degrees of *W. trilobata* invasion significantly increased soil pH values whereas high degrees of invasion did not significantly affected soil pH values. Moreover, the degree of *W. trilobata* invasion exerted significant effects on soil Ca concentration but did not significantly change other indices of soil physicochemical properties. Low and high degrees of *W. trilobata* invasion increased the richness of the soil fungal community but did not pose obvious effects on the soil bacterial community. *W. trilobata* invasion also exerted obvious effects on the community structure of soil microorganisms that take part in soil nitrogen cycling. These changes in soil physicochemical properties and community structure of soil microbial communities mediated by different degrees of *W. trilobata* invasion may present significant functions in further facilitating the invasion process.

## Introduction

Biological invasion is an important element of global change [1-4]. Invasive species have become a serious problem in the global scope because these invaders exert multiple effects on the structure and/or functions of their surrounding ecosystems [1-5]. In recent decades, ecologists have become increasingly interested in successful mechanisms of plant invasion to determine why some plants are strongly invasive while others are not [6,7]. Numerous studies have found that some plants successfully invade certain environments because these species can accelerate the succession of soil microbial communities in their rhizosphere and then strengthen the metabolic activities and community structure of the soil microorganisms to facilitate their further invasion [5,8-11]. Thus, considering the continuing increase in anthropogenic activities, the effects of plant invasion on soil microbial communities have recently received increased research interest.

Gradual succession occurs after invaders are transported from their natural habitat and progressively establish populations in invaded ecosystems [12-14]. Invasive plants exert different degrees of invasion in affected areas [14], and the community structure of soil microorganisms may be significantly affected by plant invasion along different stages of succession. Thus, understanding the effects of different degrees of plant invasion on soil microbial communities is important in elucidating the mechanism underlying the success of plant invasion. Unfortunately, existing studies on plant invasion mainly focus on the impacts of invasive plants on native ecosystems; such studies often ignore the invasion degrees of invading species or do not discuss the effects of different degrees of plant invasion on the community structure of soil bacteria and fungi.

The present study was carried out using cross-site comparisons to provide a relatively complete depiction of the responses of soil microbial communities to different invasion degrees mediated by *Wedelia trilobata*. *W. trilobata* is a creeping, mat-forming perennial herb native to the tropics of Central America, which has invaded many areas of tropics and subtropics [15,16]. It has been listed as one of the most malignant weeds listed by the International Union for Conservation of Nature and Natural Resources (IUCN) [17]. In the 1970s, *W. trilobata* was introduced to China as an ornamental and groundcover plant, and rapidly escaped from gardens to roadsides and plantations [15,16]. *W. trilobata* has become recognized as a notorious weed in southern China [15,16,18]. The characteristic such as high nutrient cycling rates [especially soil nitrogen (N)] is invoked to explain the successful invasion of *W. trilobata* [19,20]. This study aims (1) to examine the effects of different degrees of *W. trilobata* invasion on soil physicochemical properties and (2) detect the effects of different degrees of *W. trilobata* invasion on the community structure of bacteria and fungi in soil subsystems. We hypothesize that (1) increasing degrees of *W. trilobata* invasion enhance soil nutrient element concentrations (especially soil N) because invasive plants have high nutrient cycling rates, especially for N [6,8,14,19-22], and that (2) low degrees of *W. trilobata* invasion significantly increases the richness of the soil bacterial community whereas high degrees of *W. trilobata* invasion significantly increases the richness of the soil fungal community because soil microbial communities are dominated by bacteria in early succession and by fungi in late succession [23].

## Materials and Methods

### Site description

Samples were obtained from five areas, namely, Haikou (19°32'–20°05'N, 110°10'–110°41'E), Tunchang (19°08'–19°37'N, 109°45'–110°15'E), Sanya (18°09'–18°37'N, 108°56'–109°48'E), Qionghai (18°58'–19°28'N, 110°07'–110°40'E), and Danzhou (19°11'–19°52'N, 108°56'–109°46'E); all areas were located in Hainan Province, P. R. China, with an area of 35 400 km^2^ and an altitude of 1 811.6 m. The study areas feature a subtropical humid climate, with an annual mean temperature of approximately 24 °C and an annual precipitation of approximately 1 500 mm. The samples were collected from public land. No specific permissions were required to obtain samples from these locations, and details on why this area was chosen need not be provided. Ethical approval to conduct the present study was not required because we did not handle or collect animals considered in any animal welfare regulations, and no endangered or protected species were involved in our sampling or experiments.

### Experimental design

In August 2010, rhizospheric soil samples with different degrees of *W. trilobata* invasion were collected from the five aforementioned areas. One sample area was divided into three sites according to the degree of *W. trilobata* invasion, i.e., uninvaded (0%, CK), low degree (<35%, LD), and high degree (>75%, HD) using the coverage of *W. trilobata* in the invaded ecosystems. Five soil samples within an approximately 5 cm radius of *W. trilobata* rhizosphere from each invasion degree in each site were collected. A total of fifteen treatment combinations were obtained: 5 sample areas × 3 invasion degrees (the related information is shown in Table 1).

**Table 1:** Degrees of *W. trilobata* invasion in the fifteen samples used in the present study.

Invasion situation	No. of sample site	Degree of invasion	Sample area
Uninvaded	1	0%	Haikou
	4	0%	Tunchang
	7	0%	Sanya
	10	0%	Qionghai
	13	0%	Danzhou
Low-degree invasion	2	34%	Haikou
	5	10%	Tunchang
	8	6%	Sanya
	11	10%	Qionghai
	14	1%	Danzhou
High-degree invasion	3	99%	Haikou
	6	90%	Tunchang
	9	97%	Sanya
	12	90%	Qionghai
	15	92%	Danzhou

All soil samples were stored in sealed sterile bags and immediately transported back to the laboratory. The soil samples were passed through a 2 mm sieve to remove leaves, plant roots, and gravel. All soil samples from one site were homogenized by thorough mixing and then stored in a refrigerator at 4 °C for further processing. Sieving and homogenization steps were carried out to decrease the discrepancies brought about by the inhomogeneity of soil contents and reduce the effects of serendipitous foreign materials on parameter determination.

### Determination of soil physicochemical properties

Soil pH values were measured using a glass electrode (1:5 soil–water ratios) after shaking the samples for approximately 30 min to equilibrate [24]. Soil moisture was determined by sampling 5 g of soil and then drying it at 105 °C for 24 h to achieve a constant weight. Soil organic matter was analyzed using the method of K_2_Cr_2_O_7_–H_2_SO_4_ oxidation. Soil N concentration was determined by the Semimicro-Kjeldahl method. Soil phosphorus (P) concentration was determined using the Mo-Sb antispetrophotography method. Soil potassium (K) concentration was determined with the NaOH-melt method. The concentrations of iron (Fe), manganese (Mn), calcium (Ca), and magnesium (Mg) were determined through atomic absorption spectrophotometry.

### Determination of genetic diversity in soil microbial communities

Genetic diversity in the soil microbial communities in the rhizospheres of *W. trilobata* was analyzed by denaturing gradient gel electrophoresis (DGGE). 16S rRNA and 18S rRNA genes were amplified with the universal bacterial primers 341F/907R [25] and the universal fungal primers NS1/Fung [26,27], respectively. A 40-base pair G + C-rich sequence (GC-clamp) was attached to the 5' end of the forward primers to prevent the complete separation of the strands during DGGE. PCR amplification was performed with 25 µL of 2 × Power Taq PCR MasterMix (Invitrogen, USA), 1 μL of each primer (10 μM), 1 µL of DNA extract, and 1 µL of BSA (10 mg mL^−1^); sterile ultrapure water was used to adjust the mixture to a final volume of 50 µL. PCR amplification was run on a MyCycler thermal cycle (Bio-Rad, USA). PCR amplification of 16S rRNA was performed as follows: initial denaturation at 94 °C for 5 min, followed by 35 cycles of denaturation at 94 °C for 40 s, annealing at 55 °C for 50 s and an extension at 72 °C for 50 s, and a single extension at 72 °C for 7 min; the program was ended at 25 °C. The 18S rRNA PCR program was carried out with an initial denaturation step at 94 °C for 5 min, followed by 35 cycles of denaturation at 94 °C for 30 s, annealing at 55 °C for 30 s and elongation at 72 °C for 40 s; a final elongation step at 72 °C was performed for 7 min and the program was ended at 4 °C.

DGGE was carried out using a Dcode universal mutation detection system (Bio-Rad, USA). PCR samples (30 µL) containing approximately equal amounts of PCR amplicons were loaded onto the 1 mm thick 8% (w/v) polyacrylamide gels in 1 × TAE buffer using a denaturing gradient ranging from 30% to 80% for bacterial PCR samples and 10% to 50% for fungal PCR samples (100% denaturant was defined as 7 M urea and 40% deionized formamide). Electrophoreses were performed at 60 °C and 120 V for 12 h. After staining with SYBR Green I nucleic acid gel stain (Molecular Probes, Carlsbad, CA, USA), the gels were scanned and analyzed with QuantityOne software (version 4.5, Bio-Rad, USA).

All recognized DGGE bands were excised under UV light, and a bead beating method was applied to extract DNA from the gel slices [25]. After purification with a DNA fragment purification Kit (Toyobo, Osaka, Japan), the eluted DNA was used for re-amplification with the original primer set (without the GC clamp). PCR products were sequenced by Sangon Biotech (Shanghai) Co., Ltd. (Shanghai, P. R. China). The sequences were submitted to National Center for Biotechnology Information (NCBI, http://www.ncbi.nlm.nih.gov/) for BLAST to determine their phylogenetic affiliation, and the closest relatives were identified for phylogenetic analysis.

### Analysis

DGGE banding profiles of both bacterial community and fungal community were digitized after average background subtraction for the entire gel using QuantityOne (version 4.6.2, Bio-Rad, USA). The relative intensity of a specific band was transformed according to the sum of the intensities of all bands in a pattern [28]. Bands with relative contributions below 1% were discarded from the analysis, and the Shannon–Wiener diversity (*H*') and Pielou evenness (E_H_) indices were used to estimate the community structure of the soil microorganisms. *H*' was determined by the following equation: *H*'=-Σ*P*_i_ln*P*_i_ [29], where *P*_i_ is the importance probability of the bands in a track. P_i_ was calculated as follows: P_i_=*n*_i_/*N*, where *n*_i_ is the band intensity for individual bands and *N* is the sum of the intensities of all of the bands in a single lane [30]. *E*_*H*_ was calculated as follows: *E*_*H*_=*H*'/ln*S* [31], where *S* is defined as the band amount present in a single lane [32,33].

A phylogenetic tree of the relationship between the sequences of the predominant DGGE bands and those in GenBank determined by BLAST was created through the Neighbour-joining method using Molecular Evolutionary Genetics Analysis (MEGA, version 5.1).

All data were checked for deviations from normality and homogeneity of variance before analysis. The effects of the degree of *W. trilobata* invasion on soil microbial communities and Shannon–Wiener diversity (*H*') and Pielou evenness (*E*_*H*_) indices of soil microorganisms were determined by analysis of variances (ANOVA) with site considered as a block effect using Statistical Product and Service Solutions (SPSS, version 17.0). Statistical significance was set at *P* <0.05.

## Results

### Soil physicochemical properties

Low degrees of *W. trilobata* invasion significantly increased soil pH values (Table 2, *P* < 0.05) whereas high degrees of *W. trilobata* invasion did not significantly affect soil pH values (Table 2, *P* > 0.05). Low and high degrees of *W. trilobata* invasion increased soil moisture; the difference between the effects of high and low degrees of invasion on soil moisture was not significant (Table 2, *P* > 0.05).

**Table 2:** Physicochemical properties of the soil samples.

*Invasion situation*	*Soil pH*	*Soil moisture*	*Organic matter*	*N*	*P*	*K*	*Fe*	*Mn*	*Ca*	*Mg*
Uninvaded	4.676±0.645**^*b*^**	14.660±3.239**^*a*^**	2.224±0.784**^*a*^**	0.121±0.041**^*a*^**	0.034±0.010**^*a*^**	0.328±0.111**^*a*^**	60.680±16.235**^*a*^**	59.100±22.834**^*a*^**	1561.860±369.987^ab^	141.360±46.887**^*a*^**
Low-degree invasion	6.240±0.340**^*a*^**	17.280±2.333**^*a*^**	2.060±0.694**^*a*^**	0.108±0.037**^*a*^**	0.044±0.008**^*a*^**	0.230±0.063^ab^	48.980±12.032**^*a*^**	35.620±8.612**^*a*^**	2811.480±645.846**^*a*^**	127.780±46.290**^*a*^**
High-degree invasion	4.742±0.406**^*b*^**	17.360±3.594**^*a*^**	2.138±0.284**^*a*^**	0.113±0.014**^*a*^**	0.037±0.009**^*a*^**	0.084±0.031**^*b*^**	37.820±21.463**^*a*^**	37.240±21.072**^*a*^**	1057.800±254.297**^*b*^**	105.960±46.294**^*a*^**

The values in the table represent means of the values of the five areas with the same degree of *W. trilobata* invasion. Data with different superscript letters in a vertical row indicate significant difference (*P* < 0.05). Legends: units of soil moisture, N, P, and K are in % (W/W) and units of organic matter, Fe, Mn, Ca, and Mg are in mg kg^−1^.

Soil Ca concentration under low degrees of *W. trilobata* invasion was significantly higher than that under high degrees of *W. trilobata* invasion (Table 2, *P* < 0.05). K concentrations decreased significantly with increasing degree of *W. trilobata* invasion (Table 2, *P* < 0.05). Both low and high degrees of *W. trilobata* invasion did not significantly change soil organic matter, N, P, Fe, Mn, and Mg concentrations (Table 2, *P* > 0.05).

The ANOVA results revealed that the degrees of *W. trilobata* invasion significantly affected the soil Ca concentration (Table 3, *P* < 0.05). However, the degrees of *W. trilobata* invasion did not pose obvious effects on other indices of soil physicochemical properties (Table 3, *P* > 0.05).

**Table 3:** ANOVA of the effects of the degree of *W. trilobata* invasion on the soil physicochemical properties and Shannon–Wiener diversity (*H*') and Pielou evenness (*E_H_*) indices of soil microorganisms.

	*Soil pH*	*Soil moisture*	*Organic matter*	*N*	*P*	*K*	*Fe*	*Mn*	*Ca*	*Mg*	*H'-B*	*H'-F*	*E_H_-B*	*E_H_-F*
F	3.373	0.245	0.017	0.040	0.326	2.620	0.451	0.496	3.953	0.148	0.697	4.364	3.152	0.445
*P*	0.069	0.786	0.983	0.961	0.728	0.114	0.647	0.621	0.048*	0.864	0.517	0.038*	0.079	0.651

^*^ indicates significant differences at the 0.05 probability level. *P* values equal to or lower than 0.05 are in boldface. Legend: *H*'-B, H*'* of soil bacterial community; *H*'-F, H' of soil fungal community; *E*_*H*_-B, *E*_*H*_ of soil bacterial community; *E*_*H*_-F, *E*_*H*_ of soil fungal community.

### DGGE pattern and soil microbial communities' structure

**Figure 1:**
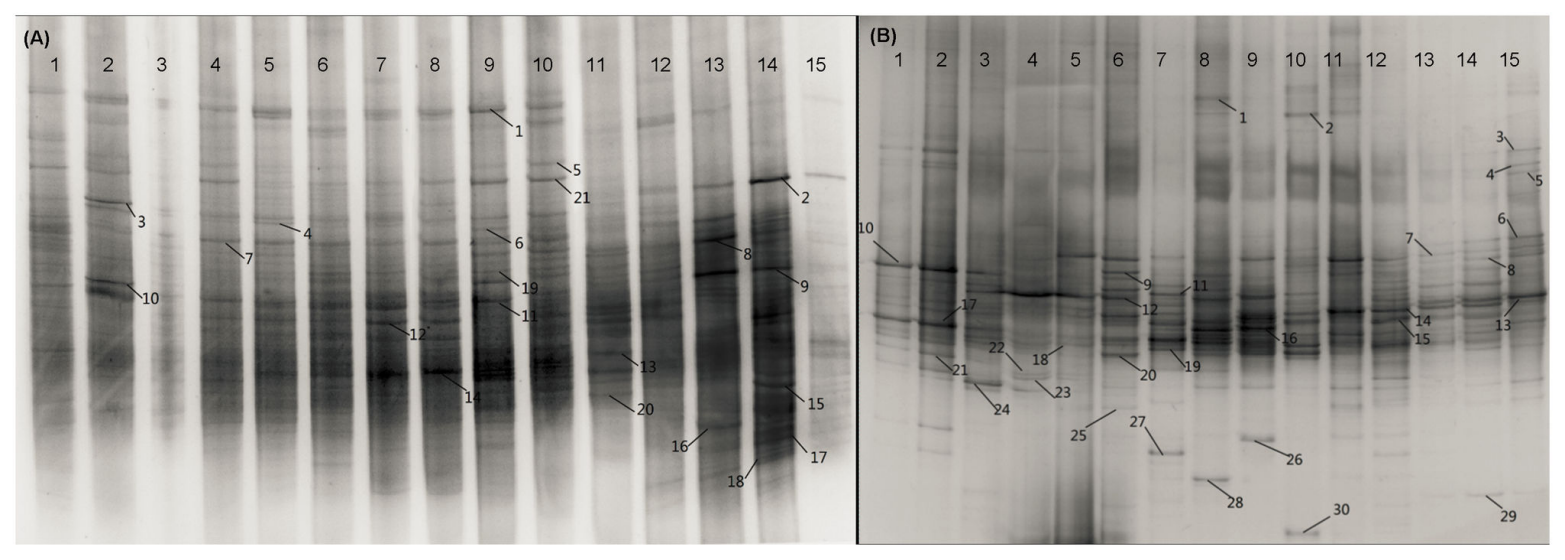
DGGE fingerprints of amplified 16S rRNA gene fragments of soil bacterial community (A) and amplified 18S rRNA gene fragments of soil fungal community (B). Straight lines indicate the DGGE bands for which the sequence was determined. Arabic numerals lies above the figure represent sample sites.

**Figure 2:**
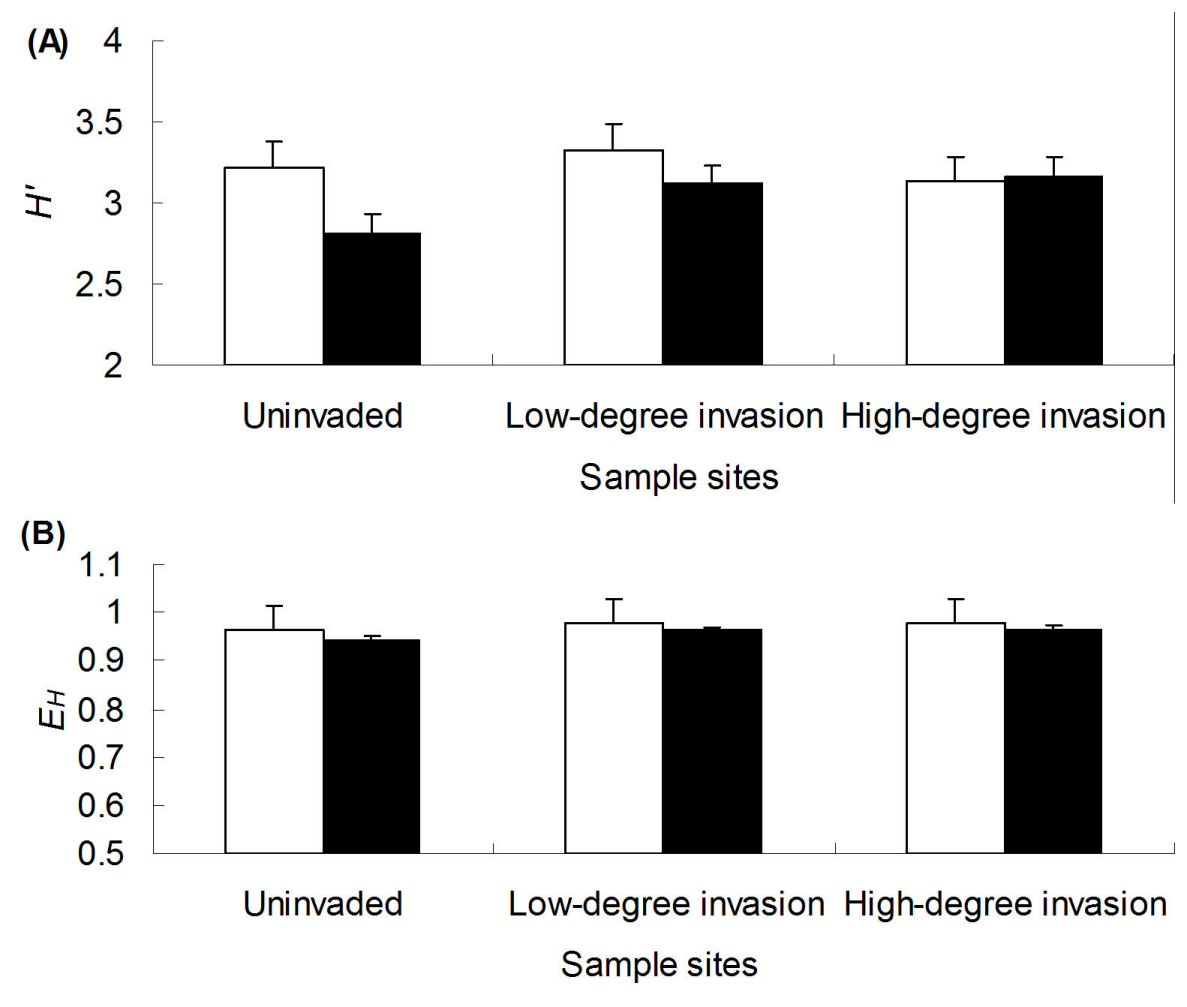
Shannon–Wiener diversity (*H*') (A) and Pielou evenness (*E_H_*) (B) indices of the soil microbial communities under different degrees of *W. trilobata* invasion. Symbols: open bars, soil bacterial community; filled bars, soil fungal community. Error bars indicate standard errors (SE, n = 3).

The community structures of soil microorganisms were compared based on DGGE analysis of 16S rRNA and 18S rRNA gene fragments. The DGGE patterns showed remarkable differences in composition among the five sample areas (Figures 1A and 1B). A significant difference was observed between Shannon–Wiener diversity of soil bacterial community and that of soil fungal community under uninvaded and low degrees of *W. trilobata* invasion but not under high degree of *W. trilobata* invasion (Figure 2A). The degrees of *W. trilobata* invasion significantly effected Shannon–Wiener diversity of soil fungal community (Figure 2A; Table 3, *P* < 0.05). However, both low and high degrees of *W. trilobata* invasion did not pose significant effects on Shannon–Wiener diversity of soil bacterial community (Figure 2A; Table 3, *P* > 0.05) or on Pielou evenness of both soil bacterial community and soil fungal community (Figure 2B; Table 3, *P* > 0.05).

**Figure 3:**
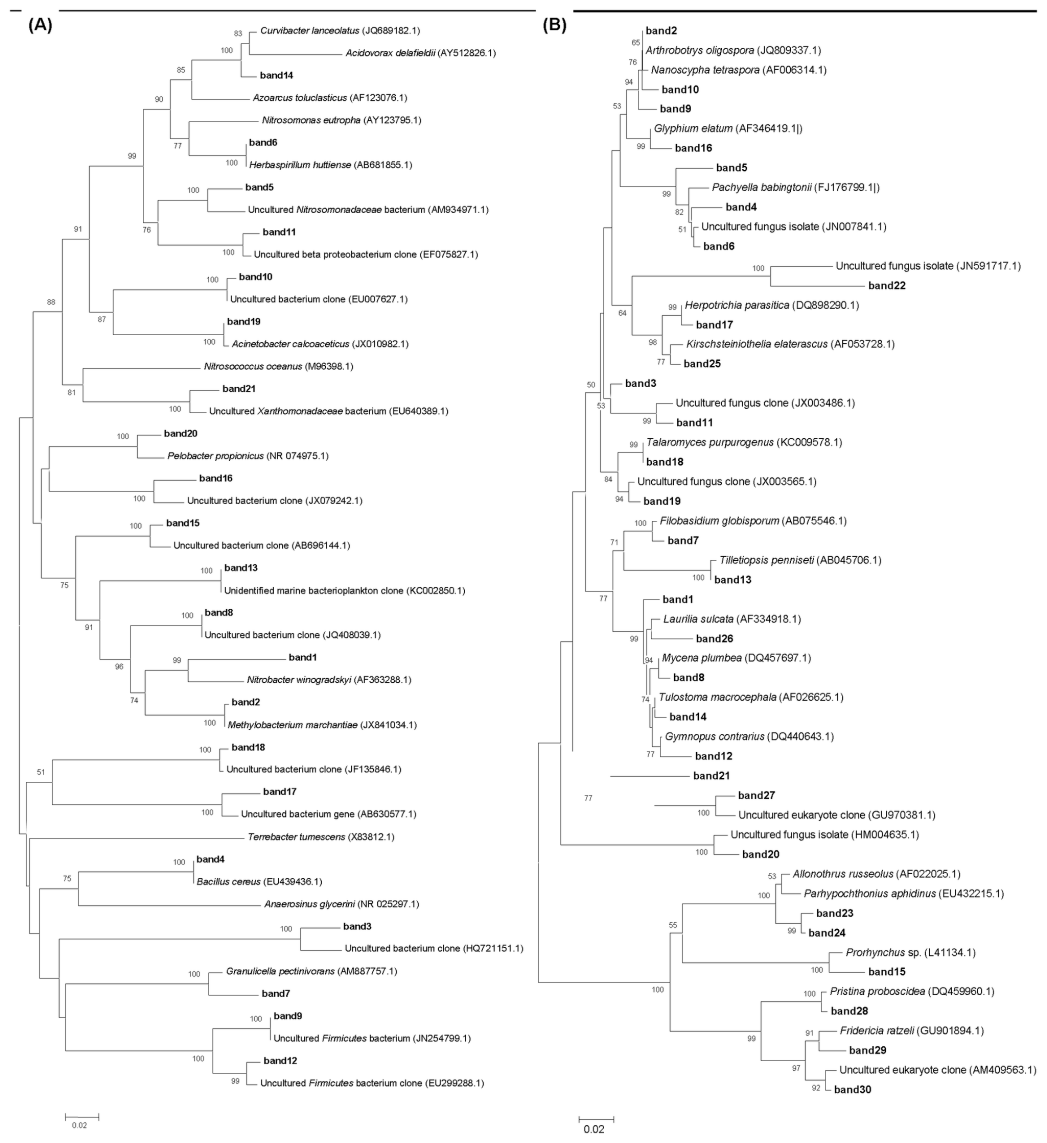
Phylogenetic tree showing the relationship of the 21 predominant 16S rRNA gene-based DGGE bands (A) and the 30 predominant 18S rRNA gene-based DGGE bands (B). Numbers at the node are bootstrap values based on 1000 re-samplings. The scale bar represents percentage similarity. Genbank accession numbers are shown in parentheses.

A total of 51 DGGE bands were sequenced, including 21 predominant 16S rRNA gene-based DGGE bands for soil bacterial community and 30 predominant 18S rRNA gene-based DGGE bands for soil fungal community (Figures 1A and 1B). The relationships of the 21 predominant 16S rRNA gene-based DGGE bands and the 30 predominant 18S rRNA gene-based DGGE bands are shown in the phylogenetic tree in Figures 3A and 3B. Obvious differences in the soil microbial communities (especially for the soil bacterial community) were observed among sites with different degrees of *W. trilobata* invasion (Figures 3A and 3B). For example, low degrees of *W. trilobata* invasion significantly increased the abundance of bands 1 and 5 of the soil bacterial community in Haikou as well as the abundance of band 5 of the soil bacterial community in Tunchang (Figures 1A and 3A). Increasing degrees of *W. trilobata* invasion increased the abundance of bands 1 and 5 of soil bacterial community in Sanya (Figures 1A and 3A). Both low and high degrees of *W. trilobata* invasion decreased the abundance of band 5 of the soil bacterial community in Qionghai (Figures 1A and 3A). Bands 1 and 5 of the soil bacterial community were respectively identified as Nitrobacter and Nitrosomonadaceae through BLAST (Figure 3A).

## Discussion

### Soil physicochemical properties

Previous studies have shown that plant invasion significantly elevates soil pH values [34-37]. This result may be mainly attributed to the fact that invasive plants have high nitrate uptake rates, which elevate soil pH values because the decrease in soil nitrate are known to elevate soil pH values [34,38]. Similar values are only obtained under low degrees of *W. trilobata* invasion in the present study. High degrees of *W. trilobata* invasion did not significantly affect soil pH values. In previous studies, the metabolic activities and community structure of soil microorganisms were highly correlated with soil pH values [39-41]. Thus, we believe that changes in soil pH values mediated by low degrees of *W. trilobata* invasion can enhance the succession of soil microbial communities in the rhizosphere and facilitate further invasion. Changes in soil pH values may play a minor role in the invasion process under high degrees of *W. trilobata* invasion.

Soil moisture is an important driver of plant invasion [42,43]. Many studies have revealed a positive correlation between soil moisture and the degrees of plant invasion [44,45]. Other researchers have found that invasive plants positively affect soil moisture in the invaded ecosystem [37,46] and that soil moisture is a major factor that influences the metabolic activities and community structure of soil microorganisms [47-49]. Therefore, changes in soil moisture induced by invasive plants can affect the changes in soil microbial communities in the rhizosphere and enhance plant invasiveness. However, in the present study, both low and high degrees of *W. trilobata* invasion did not significantly affect soil moisture. This result indicates that *W. trilobata* invades ecosystems via pathways other than through soil moisture changes.

Accumulated evidence suggests that invasive plants have high rates of nutrient cycling, especially for N [6,8,14,19-22], and higher soil P availability is often correlated with the invasion degrees of plants [50,51]. Thus, we hypothesize that *W. trilobata* invasion can enhance soil nutrient element concentrations (especially soil N and P) with increasing invasion degree. Differing from our initial hypothesis, however, the results of the present study showed that both low and high degrees of *W. trilobata* invasion did not exert significant effects on soil N, soil P, and soil organic matter concentrations. This result is consistent with a previous study [52] that found neutral effects of plant invasion on soil N or P. Other study [53] found no difference in soil N concentrations with and without the invasion by *Phalaris arundinacea* in wet prairie vegetation. The neutral effect of plant invasion on soil N may be because of the compensation of increased N demand with increased N supply [54]. As such, we believe that *W. trilobata* invades ecosystems via pathways other than through high rates of nutrient cycling.

### Structure of soil microbial communities

Several studies have shown that plants successfully invade some environments because these species can accelerate the succession of soil microbial communities in their rhizosphere and promote microbial functions, which facilitate invasion process [5,8-11]. Thus, with continuous increases in anthropogenic activities causing accelerated rates of biological invasion, considerable interest in understanding the ecological effects of plant invasion on soil microbial communities has grown [5,9,55-57]. Some investigators have suggested that invasive plants trigger the changes in the structure of biological communities in invaded ecosystems [1,13,14,58,59], especially soil microbial communities [5,9,55-57], in a way that results in positive feedback for the invading plants and negative feedback for the native plant communities [5,60-62]. Recent studies have confirmed that bacteria dominate soil microbial communities in early succession and that fungi dominate these communities in late succession [23]. Based on this finding, we hypothesized that low degrees of *W. trilobata* invasion increased the richness of soil bacterial community whereas high degrees of *W. trilobata* invasion increased the richness of soil fungal community. Results obtained in the present study are only partly consistent with our hypothesis. Both low and high degrees of *W. trilobata* invasion significantly increased the richness of soil fungal community. However, the richness of soil fungal community showed no significant difference between low and high degrees of *W. trilobata* invasion. Moreover, both low and high degrees of *W. trilobata* invasion did not exert significant effects on the richness of soil bacterial community. Thus, the results of the present study show that different degrees of plant invasion can trigger changes in the richness of soil fungal community but not in soil bacterial community. This finding indicates that soil fungal community play an important role in the invasion process of invasive plants. Changes in the soil fungal community mediated by *W. trilobata* invasion may be attributed to changes in the soil physicochemical properties after plant invasion [1,13,59]. Differences in soil characteristic may also contribute to differences in the invasion degrees of invasive plants as well as the community structure of soil microorganisms. The results of the present study are partly inconsistent with those presented in a previous study [63], which found that *Acacia dealbata* invasion can lead to significant increases in the richness of soil bacterial community and significant reductions in the richness of soil fungal community in grassland ecosystems. Differences in results may be attributed to differences in the soil physicochemical properties studied, plant species used, time span of plant invasion, and the time scale of the studies.

Invasive plants often feature faster growth rates and respond more opportunistically to nutrients (especially N) [64]. Several studies [65,66] show that the invasion degree induced by plants is positively correlated with soil nutrients (especially N). Thus, invasive plants may maximize their invasiveness by accelerating soil nutrient cycling (especially N cycling) [67-70], particularly through the changes in the community structure of functional microorganism, such as soil microorganisms that take part in N cycling (i.e., N-fixing bacteria, nitrifying bacteria, nitrosifying bacteria, ammonia oxidizing bacteria, and denitrifying bacteria) [68,69,71-74]. Low degrees of *W. trilobata* invasion significantly increased the abundance of Nitrobacter and Nitrosomonadaceae in Haikou as well as the abundance of Nitrosomonadaceae in Tunchang. *W. trilobata* invasion also increased the abundance of Nitrobacter and Nitrosomonadaceae in Sanya but decreased the abundance of these bacteria in Qionghai. This finding indicates that invasive plants show altered invasiveness through changes in community structure of soil microorganisms that take part in N cycling.

The present study sought to determine the effects of different degrees of plant invasion on soil microbial communities to better understand the mechanism of plant invasion. Different degrees of *W. trilobata* invasion can trigger changes in soil physicochemical properties. Both low and high degrees of *W. trilobata* invasion significantly increased the richness of soil fungal community but not that of soil bacterial community. Invasive plants can induce changes in the community structure of soil microorganisms that take part in N cycling. Changes in the soil physicochemical properties and community structure of soil microbial communities mediated by *W. trilobata* invasion may play an important role in facilitating further invasion.
